# Microglia Modulate Cortical Spreading Depolarizations After Ischemic Stroke: A Narrative Review

**DOI:** 10.1007/s12028-022-01469-4

**Published:** 2022-03-15

**Authors:** Kathryn N. Kearns, Lei Liu, Sauson Soldozy, Khadijeh A. Sharifi, Mark E. Shaffrey, Min S. Park, Petr Tvrdik

**Affiliations:** 1 Department of Neurosurgery, University of Virginia Health System, Charlottesville, VA USA; 2 Department of Neuroscience, University of Virginia, Charlottesville, VA USA

**Keywords:** Microglia, Calcium signaling, In vivo imaging, 2-Photon, Ischemic stroke, Iso-electric depolarizations, Cortical spreading depolarization

## Abstract

Cortical spreading depolarizations (CSDs) are characterized by waves of diminished electroencephalography activity that propagate across the cortex with subsequent loss of ionic homeostasis. CSDs have been found in many pathological conditions, including migraine, traumatic brain injury, and ischemic stroke. Because of CSD-associated ionic and metabolic disturbances at the peri-infarct area after ischemic stroke, it is thought that CSDs exacerbate tissue infarction and worsen clinical outcomes. Microglia, the main innate immune cells in the brain, are among the first responders to brain tissue damage. Recent studies demonstrated that microglia play a critical role in CSD initiation and propagation. In this article, we discuss the significance of CSD in the setting of ischemic stroke and how microglia may modulate peri-infarct CSDs, also known as iso-electric depolarizations. Finally, we discuss the significance of microglial Ca^2+^ and how it might be used as a potential therapeutic target for patients with ischemic stroke.

## Introduction

In response to brain injury, neurons undergo synchronized depolarization that initiates at the site of injury and propagates across the cortex. This phenomenon is known as a cortical spreading depolarization (CSD) [1, 2]. After a window of recovery, characterized by depression of electrical activity, neurons slowly repolarize and electrical homeostasis is reestablished. CSDs have been shown to occur after a variety of cerebral insults, including migraine, trauma, cortical chemical exposure, and ischemia [2–4]. The molecular hallmark of CSD is a near-complete breakdown of the transmembrane ion gradients, with subsequent increases in extracellular glutamate, adenosine triphosphate (ATP), and K^+^ and intracellular Ca^2+^, Na^+^, and Cl^−^ [5–7]. This phenomenon was first inferred from the examination and analysis of scotoma during migraine aura [8, 9]. The researchers surmised that visual migraine auras might be caused by electrical disturbances in the cortex and began attempting to correlate visual auras with the speed of cortical spreading depression of activity recorded by Leao, who pioneered electrophysiological measurements of CSD [8–10]. Similar waves of depolarization activity have been observed in other cranial pathologies, such as subarachnoid hemorrhage and traumatic brain injury [11, 12]. Although tissue with reversible injury can recover electrical homeostasis and resume normal function, tissue facing permanent damage, as in the setting of ischemic stroke, is unable to return to baseline, and neurologic deficits result [13, 14].

### Ischemia-Induced Mechanisms Driving Spreading Depolarizations

Ischemic stroke is characterized by vascular occlusion resulting in diminished cerebral blood flow, creating a hypoxic environment. Without sufficient levels of oxygen, ATP production decreases, energy deficit increases, and neurons within the affected tissue are unable to maintain the plasma membrane potential [15, 16]. Such an insult triggers progressive CSDs, which radiate across the cortex at a speed of 3–5 mm/s [3, 17]. Cell membrane depolarization leads to K^+^ and neurotransmitter egress out of the cell, whereas Na^+^, Cl^−^, and Ca^2+^ rapidly enter the cell [6, 7]. Because of the influx of positively charged electrolytes, neuron cell bodies begin to swell from their resulting hyperosmolar state, and higher intracellular Ca^2+^ levels trigger apoptotic processes [16]. The damaged neuron releases glutamate and free radicals into the extracellular space, which activate N-methyl-D-aspartate (NMDA) receptors on the surrounding neurons and induce further release of inflammatory cytokines [7, 15, 16]. Matrix metallopeptidases are subsequently released and contribute to the breakdown of the blood–brain barrier [18, 19]. These cumulative processes produce an acidic, hypoxic, and hyperkalemic environment that stimulates vasoconstriction, exacerbating the oxygen and energy deficits [6, 16]. This leads to a cyclical progression of neuronal damage and expansion of the ischemic tissue volume, as the peri-infarct region cannot adequately repolarize [14, 20].

### Clinical Evidence for Iso-Electric Spreading Depolarizations and Depressions

CSDs have been measured in neurocritical care patients with large middle cerebral artery ischemic strokes using subdural electrocorticography strips placed over the affected area [21, 22]. Although infarcted tissue does not exhibit electrical activity, electrocorticography strips placed over the peri-infarct region capture depressed baseline electrical activity and slow depolarization waves as ischemic time progresses [12]. CSDs are commonly seen in ischemic strokes, and more frequent depolarization events appear to be directly associated with increased infarct size [2, 4, 21, 23, 24]. Compared with CSDs, which have a normal or near-normal initial baseline function, iso-electric spreading depressions have lower baseline activity and are slower to repolarize [14]. Patients with ischemic stroke who experience a transition from CSD to iso-electric spreading depression typically have more significant post-stroke neurological deficits compared with those with sustained CSD [25].

### Microglial Roles in the Initiation and Amplification of Spreading Depolarizations

Microglia are a principal component of the cerebral immune system, which is readily activated in response to noxious stimuli. In the resting state, microglia constantly surveil the surrounding brain parenchyma with long ramified processes [26–28]. If an insult is detected, the microglia change morphology and become more amoeboid in shape to facilitate reactive responses in diverse activation states [29, 30]. Microglia may detect ischemia from signals such as damage-associated molecular patterns, elevated extracellular ATP and adenosine levels, or abnormal electrolyte concentrations [31–33]. These hypoxic microglia respond by either activating proinflammatory processes and releasing neurotoxic cytokines, such as tumor necrosis factor-α, interleukins 1β, and interferon-γ, or executing anti-inflammatory programs to encourage tissue repair and neuroprotection [34–37]. This dichotomous activity has made it challenging to clarify the role of microglia in ischemic stroke [38–41]. Remarkably, recent studies have suggested that microglia are important players in the induction and progression of CSD [41, 42]. For example, Pusic and coauthors [42] examined microglia-depleted organotypic hippocampal slice cultures and were unable to induce CSD at all. Conversely, restoration of microglia to previously depleted slice cultures enabled repeated CSD [42]. Similarly, animal studies with selectively depleted microglia in the brain exhibited diminished CSD occurrence and frequency [43]. Therefore, microglia appear to be required for CSD initiation and progression. It is somewhat surprising that microglia, which account for only about 10% of total brain cells, have such a profound impact on CSD. One important mechanism in microglia–neuron communication appears to be operating via NMDA receptors. It has been shown that NMDA receptors are required for CSD induction and propagation, and blockade of NMDA receptors inhibits CSD [44–46]. Further, Moriguchi et al. [47] have demonstrated that microglia can potentiate NMDA-receptor-mediated synaptic current in neurons. The same group showed that NMDA-receptor-mediated current in neurons increased 10-fold after application of microglia-conditioned medium [48]. This effect was mediated through activation of the glycine site on NMDA receptors by microglia secreting soluble factors [48]. Together, these findings support the notion that activated microglia affect NMDA currents and consequently increase neuronal excitability [49, 50].

### Microglial Calcium as a Therapeutic Target in Ischemic Stroke

**Fig. 1:**
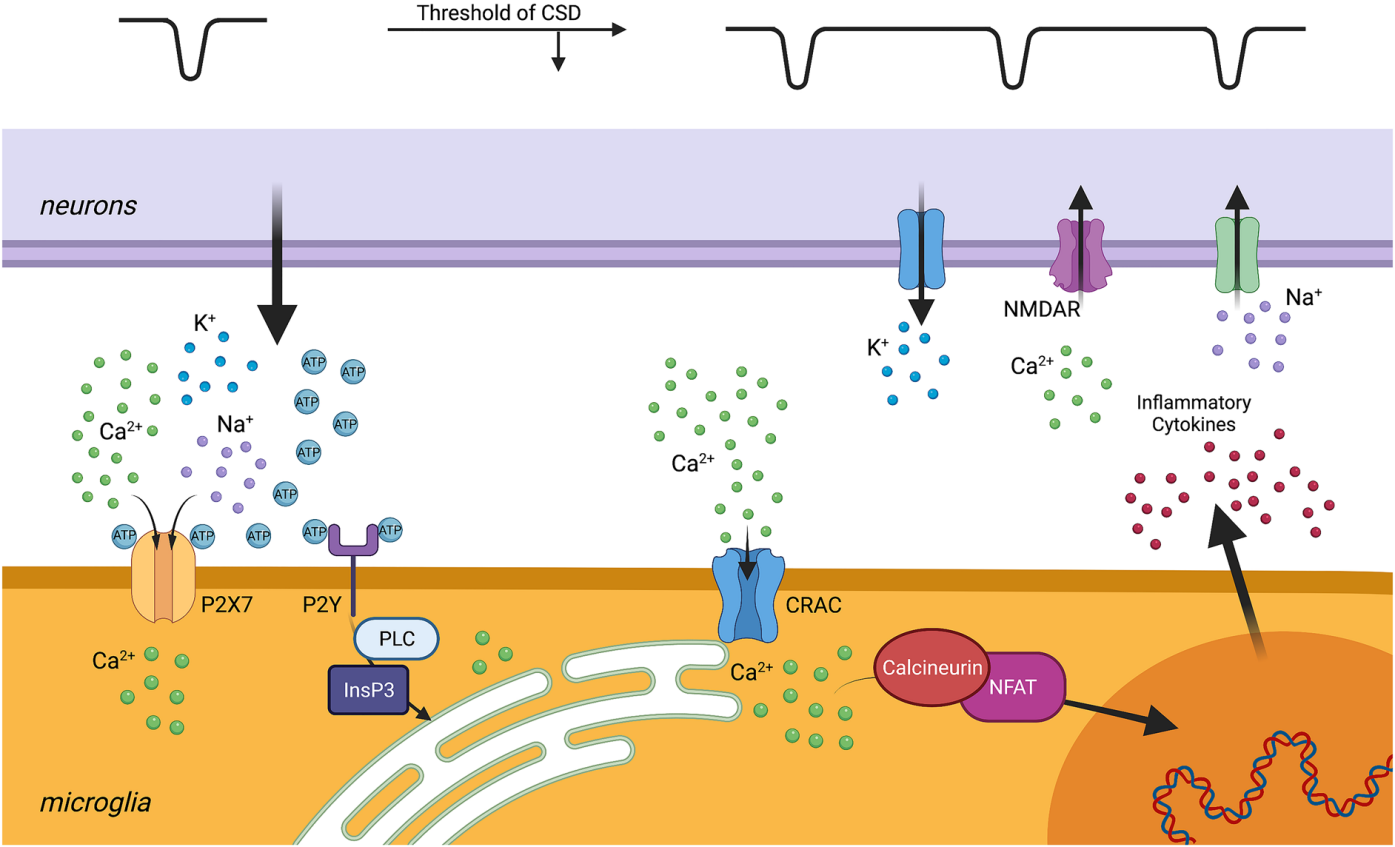
Neuron–microglia interactions in the setting of cortical spreading depolarization (CSD). After ischemic injury, neurons and microglia can engage in a self-propagating feedback loop, potentially worsening stroke outcome. Initial CSDs, occurring during prolonged depolarization of neurons at around − 10 mV, cause increase of extracellular purines, such as adenosine triphosphate (ATP), adenosine diphosphate (ADP), and adenosine, as well as potassium (K^+^). ATP/ADP activate both ionotropic purinergic receptors, P2X, and metabotropic purinergic receptors, P2Y, on microglia. Activation of P2X channels mediates Ca^2+^ influx in microglia, whereas the activation of P2Y receptors triggers Ca^2+^ release from microglial endoplasmic reticulum (ER) through phospholipase C–inositol 1,4,5-trisphosphate (PLC-IP3) signaling pathways. The depletion of ER store activates the calcium release-activated calcium (CRAC) channels, mediating additional Ca^2+^ influx into microglia. These events converge on the major elevation of intracellular Ca^2+^. High Ca^2+^ levels stimulate the inflammatory cytokine production through the calcineurin–nuclear factor of activated T cells (NFAT) pathway. Cytokines affect the CSD threshold in the nearby neurons by modulating N-methyl-D-aspartate (NMDA) currents. Sustained activation of NMDA receptors (NMDAR) further increases K^+^ leak to the extracellular space, provoking the next CSD initiation and propagation. This positive feedback loop may exacerbate neuronal damage in the periinfarct penumbra after stroke

CSD-associated depolarization of neurons causes dramatic increase of the extracellular levels of K^+^, ATP, and adenosine. Adenosine and adenosine diphosphate, produced via ATP breakdown, activate purinergic receptors on microglia, elevating intracellular Ca^2+^ levels [51–57]. The Ca^2+^ influx is at least partially mediated by the combined action of the cell surface purinergic receptors and subsequently the calcium release-activated calcium (CRAC) channels. Next, elevated Ca^2+^ levels in microglia promote expression of genes encoding several proinflammatory factors, including tumor necrosis factor α. These inflammatory factors can lower the CSD threshold in neurons by initiating the flux of charged ions through the plasma membrane. This ionic flux changes the homeostatic membrane potential, contributing to increased susceptibility for the next depolarization event [58, 59]. As discussed above, activated microglia can, for example, stimulate NMDA-receptor-mediated Ca^2+^ influx into neurons by activating NMDA receptors [48, 60]. Consequently, neuronal membrane depolarization is prolonged, ionic dyshomeostasis is further aggravated, and the initiation and propagation of CSD is amplified. Therefore, we propose that neurons and microglia in the setting of CSD engage in a self-amplifying feedback loop that can increase infarct size (Fig. 1).

Microglial purinergic receptors elevate intracellular Ca^2+^ through both ionotropic and metabotropic pathways. Upon activation, the P2X7 ionotropic receptors increase their plasma membrane channel conductance, mediating Ca^2+^ influx into the cell [37, 61–63]. The metabotropic P2Y receptors, including P2Y12, trigger inositol 1,4,5-trisphosphate (IP3) activity and allow Ca^2+^ release from the microglial intracellular stores, such as the endoplasmic reticulum [51, 64, 65]. The P2Y–IP3 pathway has been shown to contribute to microglial morphology changes, phagocytosis, chemotaxis toward the site of injury, and the formation of purinergic junctions between microglia and neurons [26, 66, 67]. These junctions appear to decrease Ca^2+^ influx into damaged neurons and have a neuroprotective effect by preventing cytotoxic edema and apoptosis [66].

**Fig. 2:**
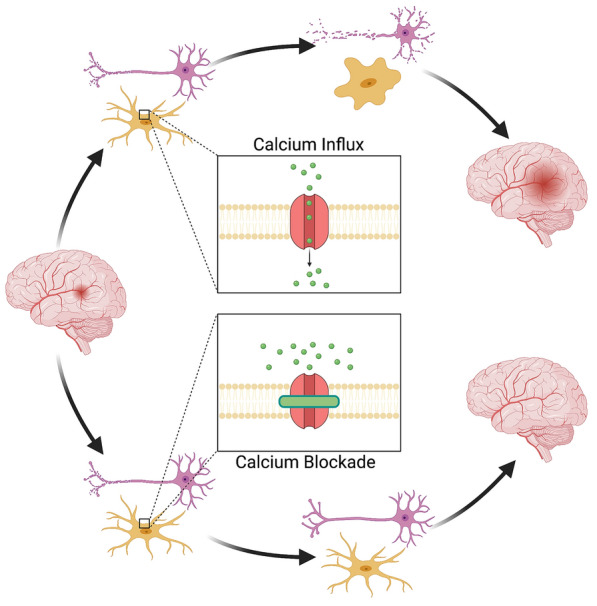
Calcium influx as an emerging treatment target for ischemic stroke. Blockade of Ca^2+^ influx through the calcium release-activated calcium (CRAC) channels may be a new therapeutic strategy for the treatment of ischemic stroke. Pharmacological inhibition of the CRAC-mediated Ca^2+^ current in the ischemic brain could facilitate significant benefits, without adverse side effects

Although this initial P2Y–IP3 pathway is initially neuroprotective and speaks to the beneficial role of microglia in neuronal recovery, as ischemic time progresses, extracellular calcium begins to enter the microglia via CRAC channels. After the intracellular Ca^2+^ stores become depleted by the initial signaling processes, the CRAC channels in the plasma membrane open to mediate a major influx of extracellular Ca^2+^ into the cell [68]. Within the hypoxic microglia, Ca^2+^ affects an incompletely understood set of downstream processes, including the calcineurin pathway, which is involved in modulating gene expression in the immune cells [69–71]. This delayed influx of calcium into microglia contributes to a persistent production of inflammatory cytokines that transitions the effect of microglia from neuroprotective to neurotoxic. A recent study by Mizuma and colleagues [69] reported the utility of the CRAC channel inhibitor, CM-EX-137, in the treatment of traumatic brain injury. They found that CM-EX-137 reduced the effect of nitric oxide and decreased intracellular microglial Ca^2+^ accumulation and the transcription of inflammatory cytokines. In their model, mice treated with CM-EX-137 after traumatic brain injury had smaller lesion sizes, less frequent hemorrhages, and improved overall neurological function compared with controls [69]. A recent study from our laboratory demonstrated that blockade of CRAC channels with CM-EM-137 partially decreased CSD-associated microglial Ca^2+^ influx [54]. Thus, CRAC channel inhibitors emerge as promising, well-tolerated, and effective antagonists of microglial activation, and prospective clinical studies are warranted to evaluate the benefits of CRAC channel inhibition in the treatment of ischemic stroke (Fig. 2).

## Conclusions

Despite the considerable amount of research on pathophysiology of ischemic stroke, effective treatments are lacking. Several therapeutic targets have been identified, but clinical validation has yet to be obtained. Currently, one of the promising targets appears to be Ca^2+^ influx in ischemic brain cells, especially in microglia. Further research is needed to fully elucidate the significance of microglial Ca^2+^ overload during the acute phase of ischemic injury, and to identify the optimal approaches to limit its harmful consequences.
